# Effective Removal of Fe and Ca From Coal Fly Ash for High‐Purity and High‐Surface‐Area Zeolite X Synthesis: A Comparative Study of Acid Refluxing and Acid Roasting Methods

**DOI:** 10.1002/open.202500467

**Published:** 2026-04-17

**Authors:** Emmanuel Monkgomotsi Victor Gaolefufa, Thapelo Manyapedza, Isaac N. Beas, Bakang F. Modukanele, Moses T. Kabomo

**Affiliations:** ^1^ Department of Chemistry University of Botswana Gaborone Botswana; ^2^ Department of Natural Resources and Materials Botswana Institute for Technology Research and Innovation Gaborone Botswana; ^3^ Department of Chemical Engineering University of South Africa Johannesburg South Africa

**Keywords:** acid leaching, coal fly ash, impurity removal, microporous zeolite X, porous materials

## Abstract

Coal fly ash (CFA) is an abundant industrial waste rich in silica and alumina and represents a viable feedstock for zeolite synthesis; however, high levels of calcium and iron limit zeolite purity and crystallisation. This study evaluates acid refluxing and acid roasting as pre‐treatment strategies for the simultaneous removal of Ca and Fe from high calcium, sub‐bituminous CFA. Acid refluxing was performed using hydrochloric acid under controlled reflux conditions, while acid roasting involved thermal treatment with hydrochloric acid in a muffle furnace. The effects of temperature (80°C–115°C), treatment time (0.5–2 h) and acid concentration (5%–37%) were systematically investigated. Both methods reduced impurity contents to below 2%, with acid refluxing achieving higher removal efficiencies (99% Ca and 97% Fe) than acid roasting (95% for both). Zeolite X synthesised from reflux‐treated CFA exhibited improved crystallinity and purity. X‐ray fluorescence spectroscopy and X‐ray diffraction analyses confirmed enhanced SiO_2_/Al_2_O_3_ ratios, while Brunauer–Emmett–Teller measurements revealed a predominantly microporous structure (∼0.6 nm) and a high specific surface area (728.8 m^2^ g^−1^), compared with mesoporous, low‐surface‐area materials obtained via acid roasting. These results demonstrate the importance of pre‐treatment in controlling zeolite properties derived from CFA.

## Graphical abstract

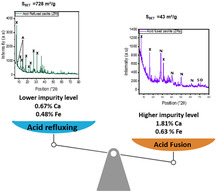

## 1 Introduction

Since the widespread adoption of electrical power, coal has accounted for the largest share of global electric power production. This trend is expected to continue due to rising petroleum and natural gas prices, especially in coal‐rich countries like Botswana [1, 2]. This reliance on coal produces large quantities of coal fly ash (CFA). Approximately 1.2 billion tons of CFA are generated annually worldwide, yet less than 5% is currently utilised due to limited technological advancement [1]. In Botswana, two power stations are currently in operation, Morupule Power Plant A and B. Together, they produce approximately 672,000 tons of CFA per year, of which less than 30% is utilised by the construction industry [1]. Most of this waste material ends up being piled in ash pits and lagoons, which are reported to be poorly managed worldwide. CFA contains harmful metals such as As, S, Pb, V, B, Cr, Al, Cd and Hg, which can contaminate water, soil and air, causing significant health issues to both humans and the environment when dumping sites are mismanaged [3]. Consequently, there is a pressing need to develop strategies to reduce CFA waste and protect the environment.

Despite containing harmful elements, CFA is also rich in aluminium (Al) and silicon (Si) oxides, which are useful for producing silicates, alumina, ceramics, glass, catalyst supports, geopolymers and zeolites [4, 5, 6]. Therefore, this waste material can be used as an inexpensive raw material for these valuable commodities, thereby reducing its environmental burden. However, impurities like Fe and Ca present in CFA adversely affect the properties and purity of these materials [2, 6]. Iron oxide reduces the optical brightness, cation‐exchange capacity (CEC) and calcium‐binding capacity of materials such as zeolites [7]. Calcium exhibits a strong affinity for Si. During zeolite synthesis, calcium forms calcium‐silicate minerals that inhibit silica (SiO_2_) hydrolysis and promote unwanted phases such as sodalite and cancrinite [8, 9]. Catalfamo et al. [8] also reported that CFAs with Ca content greater than 5% lead to hydroxysodalite or prolonged zeolite synthesis duration. Therefore, removing these impurities is necessary to synthesise high‐quality materials from CFA.

Impurities in CFA are mainly removed either by physical or chemical separation in the zeolite synthesis protocol. Physical separation uses magnetic separators to remove ferromagnetic particles, reducing Fe content to below 1% [10]. Rayalu et al. [7] achieved 75% Fe removal through a magnetic separation technique in six cycles. However, not all Fe in CFA is magnetic and is ineffective for removing nonmagnetic elements such as Ca. Consequently, physical separation alone is insufficient for complete impurity removal in one step. Chemical separation, especially using mineral acids, is more effective and cost‐efficient for removing Ca and Fe, as they are acid‐soluble [11, 12]. Hydrochloric acid (HCl) can achieve 100% calcium removal at room temperature in 5 h and significantly reduce Fe content when used at 80°C for 3 h [11]. Kongnoo et al. [13] reported a reduction in Fe content in fly ash to 0.08 g wt.% using HCl at 80°C for 3 h. Manyepedza et al. [5] achieved 99% silica purity from CFA using HCl as an impurity leaching agent after alkaline fusion. Valeev et al. [2] reported 95% Al extraction from CFA using HCl. Lv et al. [12] reported 85% and 95% Fe_2_O_3_ and CaO removal from high‐calcium‐fly ash. Apua [14] reported Fe and Ca removal of 73% and 25%, respectively, using H_2_SO_4_ under optimised conditions. Liu et al. [15]. observed extraction efficiencies of 86% and 57% Ca and Fe removal from high‐Ca‐CFA using 4 M HCl in 2 h.

Valeev et al. [2] studied the kinetics of Fe and Al leaching from CFA. They demonstrated that iron dissolution is governed by both chemical reaction and diffusion mechanisms. The reported activation energy was 33 kJ mol^−1^. Lv et al. [12] reported similar findings. However, their calculated activation energy was higher. Lv et al. [12] further revealed that the removal of both CaO and Fe_2_O_3_ impurities from fly ash is governed by layer diffusion and is characterised by similar activation energies. The similar activation energies suggest that both impurities can be removed simultaneously through controlled acid leaching. Volli et al. [6] employed a refluxing method to reduce the Ca and Fe content in CFA by 80% and 60%, respectively, enabling them to achieve high crystalline zeolite A and X. The zeolite X was reported to have high catalytic conversion (85%) of mustard oil to biodiesel. Gjyli and Korpa [16] were able to remove 74% of Ca from coal fly, which enabled the synthesis of pure zeolite X with high catalytic activity for alkylation of phenol to phenetole (Ethyl phenyl ether). Rayalu et al. [7] reported that better zeolite properties, such as crystallinity and calcium‐binding capacity, can be obtained when fly ash is acid‐treated compared to untreated fly ash. Liu et al. [17] obtained high crystalline zeolite A with a high ammonium absorption capacity of 27.5 mg/g by incorporating acid refluxing of fly ash before alkaline fusion hydrothermal step.

Previous research on zeolite synthesis from CFA has focused predominantly on the removal of Fe. Comparatively little attention is given to Ca, despite its significant impact on zeolite purity and prolonging synthesis duration [8, 18, 19]. In response, this study aims to develop a robust protocol for the simultaneous removal of both Ca and Fe from CFA. Specifically, we examined the effectiveness of two acid leaching techniques, acid refluxing and acid roasting, using HCl under varied pre‐treatment conditions. The influence of temperature, acid concentration and contact time was independently evaluated for each method. Importantly, this work is the first systematic comparison of these techniques applied to high calcium, sub‐bituminous CFA. This fly ash exhibits distinct mineralogical characteristics due to limestone injection during combustion. Finally, the suitability of the pre‐treated fly ash for zeolite synthesis is evaluated. Emphasis is placed on phase purity, crystallinity and BET surface area of the synthesised zeolite.

## 2 Experimental Methods

### 2.1 Materials and Reagents

CFA utilised in this work was obtained from Morupule Power Plant B situated in Palapye, Botswana. The chemical reagents, NaOH pellets (97%), HCl (37%) and paraffin oil, were sourced from Sigma–Aldrich and used as received.

### 2.2 Preparation and Pre‐treatment of Coal Fly Ash

The collected fly ash was first sieved to 200 microns to remove stones and large particles. The ash was then dried in an oven at 110°C overnight before treatment. Two pre‐treatment methods were evaluated for calcium and iron removal. These methods, acid roasting and acid refluxing, were systematically compared for efficiency. The influence of acid concentrations, treatment temperatures and contact time was investigated independently. For acid roasting, approximately 5 g of pre‐dried fly ash was mixed with HCl (37%) acid. The mixture was then placed in a muffle furnace and heated to 80°C–115°C for 1 h. After roasting, the treated ash was washed with distilled water and dried overnight at 90°C. Subsequently, the temperature with the highest Fe removal was selected to investigate the effects of contact time (0.5–2 h) and acid concentration (5%–37%). Samples treated by acid roasting were labelled ARs. For the acid refluxing method, the same experimental parameters were applied. However, the treatment was conducted in a reflux system rather than an open furnace. The heat was supplied using paraffin oil on a hot plate equipped with magnetic stirring. This configuration allowed continuous condensation of acid vapour and improved solid–liquid contact. The samples treated by acid refluxing were labelled acid refluxing (ARf).

### 2.3 Preparation of Zeolite Synthesis

To assess the effectiveness of different pre‐treatment methods, zeolite synthesis was performed using optimised conditions from each technique. The synthesis protocol followed the procedure reported by Gaolefufa et al. [20] In a typical experiment, pre‐treated CFA was mixed with sodium hydroxide at a weight ratio of 1.2 (NaOH: CFa). This mixture was placed in a muffle furnace and heated at 500°C for 2 h to produce water‐soluble sodium aluminosilicates. After cooling, the fused product was ground into a fine powder. The powder was then mixed with distilled water at a ratio of 7 (fused fly ash to water, FFa: H_2_O) in a borosilicate glass bottle. The slurry was then placed in a conventional oven and heated at 90°C for 12 h to allow zeolite crystals to form. Following synthesis, the solid product was filtered and repeatedly washed with distilled water until the filtrate reached pH 9. The filtered solid was then dried overnight in an oven. The resulting zeolites were labelled ZRs, ZRf and UZ according to the pre‐treatment method.

### 2.4 Material Characterisation

The bulk chemical composition of the raw and synthesised materials was determined using X‐ray fluorescence (XRF) spectroscopy (Bruker S8 Tiger). The removal efficiencies of oxides of Fe (*R*_FeO2O3_) and Ca (*R*_CaO_) from CFA by HCl were calculated using Equation (1) adopted from LV et al. [12] based on XRF chemical results of each acid treatment condition. The loss of Al_2_O_3_ from CFA during the acid pre‐treatment was also monitored using the SiO_2_/Al_2_O_3_ ratio (SAR) of the samples obtained from XRF based on Equation (2). SiO_2_ has been noted to have high stability under acidic conditions; hence, it was used as an internal standard for quantification of the chemical composition of different minerals, which have gone through acid leaching. Similarly, fly ash SiO_2_ was treated as an internal standard in this work.

X‐ray diffraction (XRD) analysis was conducted using a PANalytical Empyrean diffractometer with Cu Kα radiation. Diffraction patterns were recorded over a 2*θ* range of 5°–80°, with a step size of 0.067° at 25°C. Crystal phases were identified using international centre for diffraction data powder diffraction file (ICDD‐PDF) cards via HighScore software. Furthermore, scanning electron microscopy (SEM) was employed to examine the morphological properties of the raw CFA. The powders were dispersed in absolute isopropyl alcohol overnight to homogenise. The suspension was mounted on aluminium stubs and air‐dried. Before imaging, the samples were coated with a thin gold layer to improve electrical conductivity. Thermogravimetric analysis (TGA) was used to determine the unburnt carbon content of the CFA. A two‐atmosphere method was employed, using nitrogen for inert conditions and oxygen for oxidative conditions [21]. This approach allows separation of carbon oxidation from other thermal events, such as the decomposition of carbonates and the loss of bound water. This helps obtain an accurate estimate of the unburnt carbon in fly ash compared to one atmosphere loss on ingnition (LOI), which overestimates it. Functional groups of zeolites were identified using Attenuated Total Reflectance Fourier transform Infrared (Mettler Toledo ATIR‐FTIR).

(1) $$R(x)=1-\frac{(W(x/\mathrm{Si})p}{(W(x/\mathrm{Si})a}$$

(2) $$\mathrm{SAR}=\frac{(W(\mathrm{Si})p}{(W(\mathrm{Al})p}$$

Where *x* refers to either Al_2_O_3_, CaO or Fe_2_O_3_ in the fly ash, *R*(*x*) refers to the removal efficiency of the respective oxide by HCl acid, *W*(*x*/Si)*p* and *W*(*x*/Si)*a* are the weight ratios of the oxide of interest and Si in the acid‐treated product and original fly ash, as determined by XFR, respectively.

## 3 Results and Discussion

### 3.1 Characterisation of Raw Coal Fly Ash

**FIGURE 1:**
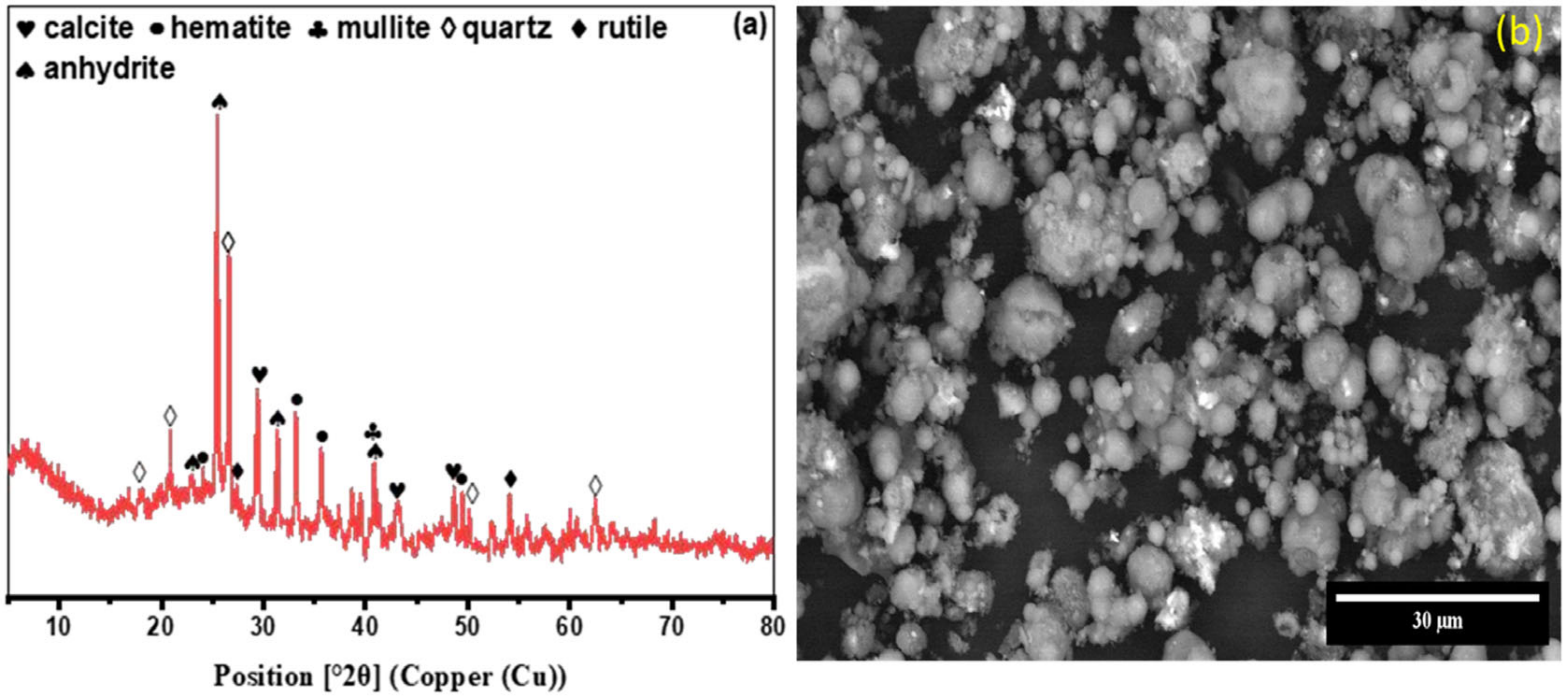
XRD diffraction pattern (a) and SEM image of untreated coal fly ash (b).

The CFA used in this study contains SiO_2_, Al_2_O_3_, CaO, Fe_2_O_3_, SO_3_, TiO_2_ and MgO as the principal oxides, with other oxides present in amounts below 0.1%, as detailed in Table 1. The X‐ray diffraction (XRD) analysis and corresponding mineral composition are presented in Figure 1a and Table 1. The mineralogical study shows that the untreated CFA has mullite (3Al_2_O_3_.SiO_2_), quartz (SiO_2_), calcite (CaCO_3_), anhydrite (CaSO_4_), haematite (Fe_2_O_3_) and rutile (TiO_2_). Despite the high concentrations of SiO_2_ (32.86%) and Al_2_O_3_ (27.2%) in the fly ash, the XRD analysis, Figure 1a and Table 1, reveals that calcium‐based minerals, calcite and anhydrite (totalling 51.4% of crystalline material), are more prominent than SiO_2_ and Al_2_O_3_ minerals like quartz and mullite (totalling 36.7%).

**TABLE 1:** Chemical and mineral composition of coal fly ash (wt.%).

SiO_2_	Al_2_O_3_	CaO	SO_3_	Fe_2_O_3_	TiO_2_	MgO	Na_2_O	P_2_O_5_
32.86	27.2	17.34	8.78	8.08	2.09	1.40	0.84	0.42
K_2_O	MnO	BaO	SrO	Cl	ZrO_2_	CuO	Cr_2_O_3_	Class
0.42	0.17	0.14	0.11	0.05	0.04	0.03	0.03	C
Anhydrite	Mullite	Calcite	Quartz	Rutile	Haematite	—	—	—
32.5	19.9	18.9	17	0.5	10.6	—	—	—

This disparity suggests that a significant portion of the SiO_2_ and Al_2_O_3_ in the fly ash exists in an amorphous state, indicated by a broad hump observed below 15°–30° 2*θ* in the diffraction pattern [22]. Amorphous phases lack sharp diffraction peaks, which explains why these oxides are less detectable than crystalline calcium minerals. It is also worth noting that the minerals of Ca and Fe make up to 62% of the crystalline material, which indicates the difficulty of achieving pure zeolite crystals using a one‐pot zeolite synthesis protocol with this fly ash [8, 9, 18]. Morphologically, the untreated CFA consists of hollow or solid spherical particles with the latter being predominant, as depicted by the SEM image (Figure 1b). This is consistent with the description of fly ashes by Dere et al. and Sivalingam et al. [4, 22].

The chemical and mineralogical composition of CFA is influenced by the type of coal burned, the combustion process, and the cooling methods used. CFAs from sub‐bituminous and lignite coals typically contain higher levels of CaO, MgO and SO_3_, and lower levels of SiO_2_ and Al_2_O_3_ compared to those from bituminous and anthracite coals. According to the American Society for Testing and Materials (ASTM C618), CFA is classified into two categories: Class C and Class F [22, 23]. Class F CFA, typically derived from bituminous and anthracite coals, contains over 70% combined SiO_2_, Al_2_O_3_ and Fe_2_O_3_, with CaO levels below 5%. In contrast, Class C CFA, which comes from lignite coals, has a higher CaO content, reaching up to 20%, and contains more than 50% combined SiO_2_, Al_2_O_3_ and Fe_2_O_3_. Based on these classifications, Morupule CFA, which contains 17.34% CaO and a combined 68.14% of SiO_2_, Al_2_O_3_ and Fe_2_O_3_, can be categorised as Class C and as sub‐bituminous CFA. Additionally, due to its high calcium content, it can also be classified as high calcium or low acid calsialic CFA [12, 24]. The elevated CaO content in Morupule CFA is attributed to the introduction of limestone into the coal combustion system to curb sulphur emissions. This also likely accounts for the significant presence of calcite and anhydrite crystals observed in the XRD diffraction pattern of the raw fly ash.

**FIGURE 2:**
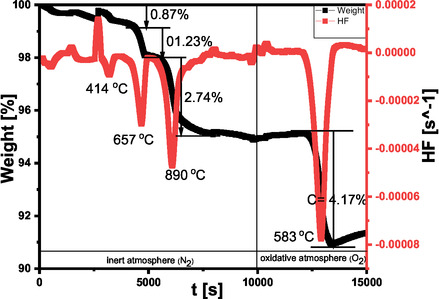
TGA and differential thermal analysis (DTA) of untreated coal fly ash.

To better understand the properties of fly ash, especially the amount of unburnt CFA, a TGA was conducted under inert and oxidative conditions. The thermal analysis plot (Figure 2) shows the temperature points at which the weight loss in the fly ash occurred, as depicted by the two‐atmosphere thermogram of the CFA. From the thermogram, weight losses of 0.94%, 4.45% and 9.21% are observed under inert (N_2_) conditions at 439°C, 693°C and 879°C, respectively. In contrast, under oxidative conditions, only one peak is observed at 533°C, with a weight loss of 3%. The loss at 439°C can be attributed to dehydration and the loss of organic volatile compounds, previously adsorbed by fly ash [21, 25]. The loss at 693°C might be due to the decomposition of carbonate minerals, while the 879°C peak might be due to the reduction of haematite by unburnt carbon in the fly ash or further decomposition of carbonate mineral calcite, as both minerals are present in the raw CFA at substantial amounts (Figure 1a and Table 1) [26, 27]. This agrees with the results of Mohebbi et al. [21], who observed the loss of water and decomposition of carbonate minerals in fly ash at a temperature range of 500°C–750°C in inert condition The reduction of haematite has also been observed to occur concurrently with the decomposition of calcite at a temperature above 750°C under the inert condition by Payá et al. [26] The single peak observed in the oxidative conditions is due to the presence of unburnt organic carbon (5.09%) in the fly ash [21], which attests to the existence of fixed organic carbon in CFA together with inorganic compounds. The reactions in both inert and oxidative reactions are described by Equations (3) – (5).

(3) $${\mathrm{Fe}}_{2}O_{3(s)}\left(\text{Hematite}\right)+C\to \mathrm{FeO}+{\mathrm{CO}}_{2(g)}$$

(4) $${\text{CaCO}}_{3\left(s\right)}\left(\text{calcite}\right)\to \mathrm{CaO}+{\mathrm{CO}}_{2(g)}$$

(5) $$C_{\left(s\right)}\left(\text{fixed organic carbon}\right)+O_{2}\to {\mathrm{CO}}_{2(g)}$$

### 3.2 Acid Pre‐Treatment of Coal Fly Ash

In the synthesis of zeolite from CFA, it is standard practice to clean the fly ash before mixing it with alkali activators to prevent the formation of unwanted phases [15]. Fly ash contains various cationic and anionic species that are soluble under alkaline environments, leading to a multi‐component system in the synthesis solution [8]. These charged species compete to direct the formation of specific crystal structures, and their influence on the solution's pH can promote the formation of undesirable crystals or a mixed crystalline product [8]. While many cationic and anionic species are soluble in neutral conditions, elements like calcium and iron are only soluble in acidic environments [12, 15]. Therefore, effective removal of these oxides requires the use of a suitable acid under optimal conditions to minimise production costs. Research has shown that mineral acids are more effective leaching agents than organic acids, which are less efficient, more expensive and more prone to retention in fly ash residue [12].

Among mineral acids, sulphuric acid is generally not recommended because it causes calcium to precipitate as CaSO_4_ on the fly ash surfaces, rather than leaching it into the solution, unlike hydrochloric and nitric acids [11]. However, nitric acid is less effective at removing elements such as vanadium, chromium, nickel and iron from fly ash. HCl is often preferred because it effectively removes most unwanted and toxic elements, is easy to handle and is cost‐effective [11]. For these reasons, this study focused on optimising the conditions for the effective removal of Fe and Ca from CFA using HCl, applied through both acid refluxing and acid roasting (roasting) methods. These techniques were systematically compared under controlled conditions, with particular attention to the behaviour of high calcium, sub‐bituminous CFA, which presents distinct mineralogical challenges. The dissolution mechanisms of Ca and Fe minerals during HCl treatment are illustrated in Equations (6) – (9). Loss of Al could also be experienced**,** as it can be dissolved by mineral acids if it exists in the amorphous state in fly ash. In the context of zeolite synthesis, the dealumination and removal of impurities from fly ash are considered beneficial, as they enhance the material's reactivity during the alkaline activation step. This, in turn, improves the catalytic properties of the resulting zeolite by increasing its activity, thermal stability and acidity [6].

(6) $${\mathrm{Fe}}_{2}O_{3\left(s\right)}\left(\text{Hematite}\right)+6H_{\left(\mathrm{aq}\right)}^{+}\to 2{\mathrm{Fe}}_{\left(\mathrm{aq}\right)}^{3+}+3H_{2}O_{\left(l\right)}$$

(7) $${{\mathrm{CaCO}}_{3}}_{\left(s\right)}(\mathrm{calcite})+{2H}_{\left(\mathrm{aq}\right)}^{+}\;\to \;{\mathrm{Ca}}_{\left(\mathrm{aq}\right)}^{2+}\;+\;{{\mathrm{CO}}_{3}^{2‐}}_{\left(\mathrm{aq}\right)}$$

(8) $${\mathrm{CaSO}}_{4(s)}(\mathrm{anhydrite})+2H_{\left(\mathrm{aq}\right)}^{+}\to {\mathrm{Ca}}_{\left(\mathrm{aq}\right)}^{2+}+{\mathrm{SO}}_{4(\mathrm{aq})}^{2‐}$$

(9) $${\mathrm{Al}}_{2}O_{3\left(s\right)}\left(\text{amophous}\right)+6H_{\left(\mathrm{aq}\right)}^{+}\to 2{\mathrm{Al}}_{\left(\mathrm{aq}\right)}^{3+}+3H_{2}O_{\left(l\right)}$$

### 3.3 Effect of Temperature

**FIGURE 3:**
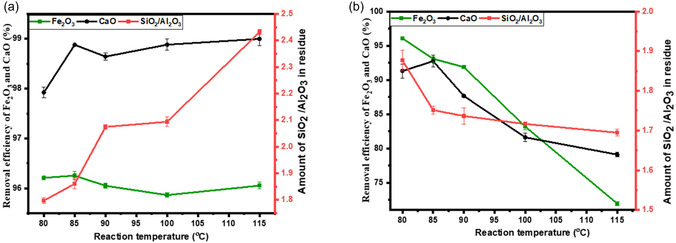
Effect of reaction temperature on Ca and Fe removal under (a) acid refluxing conditions: HCl acid, *L*/*S* (2 ml/g), 1 h and (b) acid roasting conditions: HCl acid, *L*/*S* (2 ml/g), 1 h.

According to collision theory, an increase in temperature increases the kinetic energy of molecules and their collision frequency. This enhances the interaction between acid ions and fly ash particles, leading to higher extraction efficiencies as more molecules can easily overcome the activation barrier. In the acid refluxing system (Figure 3a), the removal efficiency of both Ca and Fe increases with temperature up to 85°C, then reaches a constant value for Ca. For Fe, the removal efficiency drops as the temperature exceeds 85°C. These results are consistent with those of Lv et al. [12], who observed a decrease in removal efficiency as reaction temperature increased from 80°C to 90°C under reflux conditions. It should also be noted that leaching impurities from fly ash with HCl is accompanied by a loss of Al, as evidenced by the increase in the Si/Al ratio of the treated samples. For this parameter, 80°C was adopted, as there is a more than 1% increase in efficiency between 80°C and 85°C, to reduce energy consumption.

The increase in temperature was also found to increase Al loss, reaching up to 50%, as indicated by the SAR of 2.43 at 115°C (Figure 2a). Lv et al. [12] similarly reported excessive dealumination at elevated temperatures. The observed increase in the SAR with pre‐treatment is primarily attributed to the preferential leaching of Al_2_O_3_, since SiO_2_ remains relatively stable under acidic conditions. While the removal of other acid‐soluble impurities such as CaO and Fe_2_O_3_ may increase the relative abundance of SiO_2_ in the bulk composition, it does not directly impact the SAR, which is governed by the balance between these two oxides. Volli et al. [6] also observed an increase in the SAR of fly ash after HCl acid treatment, rising from 2.44 to 2.75 wt.%. This was accompanied by a significant loss of CaO, which decreased from 7.93 to 1.5 wt.%. They attributed these changes to the dissolution of Ca, Al and Fe in the fly ash, leading to an increase in the relative weight of other oxides, such as SiO_2_. Based on these findings, it is recommended to avoid using high temperatures for the pre‐treatment of CFA intended for the extraction of Al and for synthesising silica‐lean zeolites from fly ash.

On the contrary, an increase in temperature has adverse effects in the acid roasting system, as it lowers the extraction efficiencies of Fe and Ca. For instance, 91% and 96% removal efficiencies of Fe and Ca were achieved at 80°C, whereas 115°C gave 79% and 72%, respectively, as depicted in Figure 3b. The reduction in HCl removal efficiency in this system may be attributed to the increased loss of HCl caused by its high evaporation rate at elevated temperatures [28]. Another possible explanation is the transformation of aqueous HCl, which is fully ionised at ambient temperatures, into molecular HCl, which becomes predominant at higher temperatures [11, 28]. In either case, the contact between the acid and fly ash particles is reduced, limiting the diffusion of H^+^ ions. Zhang and Itoh [11] observed that while complete removal of calcium from CFA can be achieved using HCl at ambient temperature, the removal efficiency decreases to 77% as the temperature rises. Similarly, Bai et al. [28] reported evaporation of sulphuric acid at elevated temperatures during the development of a process to extract aluminium from CFA via thermal decomposition, thereby reducing extraction efficiency.

### 3.4 Effect of HCl Acid Concentration

**FIGURE 4:**
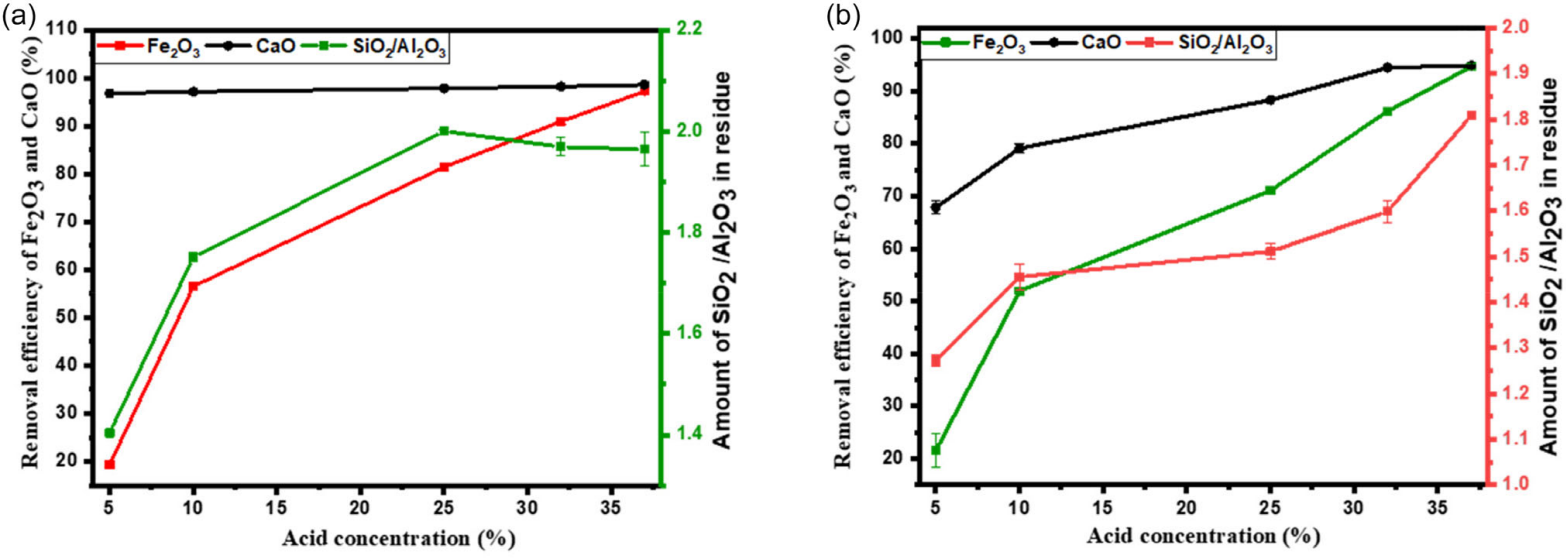
Effects of acid concentration on removal efficiency of Ca and Fe using (a) acid refluxing conditions: HCl acid, *L*/*S* (2 ml/g), 1 h, 80°C and (b) acid roasting conditions: HCl acid, *L*/*S* (2 ml/g), 1 h, 80°C.

HCl acid dissociates into proton/hydronium (H_3_O^+^) and chloride (Cl^−^) ions when in solution. It is the hydronium ion that is believed to be an agent that is responsible for interacting with the basic iron oxides and calcium minerals and solubilising them into Ca^2+^ and Fe^3+^ ions (Equations (6) – (9)) [12]. Increasing the acid concentration increases the concentration of hydronium ions, thereby accelerating the hydrolysis of basic compounds in the fly ash particle and achieving a high extraction efficiency. An increase in HCl acid concentration from 5% to 37% improves the removal efficiency of Fe from CFA, rising from less than 19% to nearly 95% under both acid reflux and acid roasting systems, as shown in Figure 4a,b. For Ca, the removal efficiency increases with acid concentration in the acid roasting system. In contrast, the acid reflux system achieves a high removal efficiency of approximately 96% even at the lowest tested concentration (5% HCl), with only marginal improvement at higher concentrations. This contrast highlights that Fe removal is strongly dependent on acid strength in both systems, while Ca removal is highly efficient under reflux conditions regardless of acid concentration.

The higher acid concentration required for effective removal in the acid roasting system is due to the evaporation of HCl, which serves as both the solvent and the leaching agent. In the open‐roasting setup, significant HCl loss occurs at elevated temperatures, reducing the availability of H^+^ ions to react with fly ash components, as much of the acid shifts into the vapour phase. In contrast, the reflux system continuously condenses HCl vapour back into the liquid phase, maintaining a stable acid concentration throughout the process – an advantage previously noted by Zhang and Itoh [11]. Additionally, the reflux setup provides better acid‐solid contact through continuous stirring, unlike the static conditions in acid roasting. It is also observed that higher acid concentrations in both systems lead to greater aluminium loss due to lower solution pH, thereby enhancing the leaching of Al_2_O_3_ from the fly ash matrix [12].

### 3.5 Effects of Acid Leaching Duration

**FIGURE 5:**
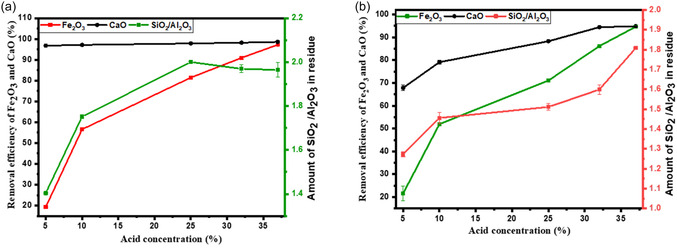
Effects of acid leaching duration on removal efficiency of Ca and Fe using (a) acid refluxing conditions: 37% HCl(aq), *L*/*S* (2 ml/g), 80°C and (b) acid roasting conditions: 37% HCl(aq), *L*/*S* (2 ml/g), 80°C, 37%.

Prolonged treatment gives the acid more time to interact with the fly ash particles, which not only dissolves impurities on the surface but also reaches those within the core of the fly ash. This enhances the removal of Ca and Fe impurities from the CFA. The leaching process is believed to be controlled by both chemical reaction and diffusion [2, 12]. Upon first contact, the acid dissolves Ca and Fe on the ash surface, forming micropores and corrosion‐induced cracks. The H^+^ ions then diffuse through these openings, reaching and dissolving the core elements of the CFA particles, such as Al [12]. The removal efficiency of Fe and Ca improves with longer reaction times in both acid refluxing and acid roasting systems, with acid refluxing showing better overall performance. In the acid refluxing system (Figure 5a), Fe removal increases steadily and stabilises at approximately 96%, while Ca removal reaches just above 98% and remains consistent beyond 90 min. In the acid roasting system (Figure 5b), Fe removal reaches around 95% after 60 min, but extending the reaction time further results in a slight decline in efficiency. For Ca, the removal efficiency increases slightly, from approximately 0.4% at 30–120 min under the acid refluxing system, reaching just above 98%. In contrast, under the acid roasting system, Ca removal decreases slightly after 60 min, stabilising at around 92%. The SAR shows a general upward trend across both systems, with final values of 2.18 for acid roasting and 2.37 for acid refluxing at 120 min. These increases are primarily due to aluminium leaching from the fly ash during prolonged reaction times. Prolonged leaching enhances the removal of impurities and facilitates the diffusion of H^+^ ions deeper into the fly ash particles, exposing more aluminium minerals to HCl and contributing to the observed changes in the SAR [12]. Similar observations were made by Liu et al. [15], who reported a 25% loss of Al_2_O_3_ when the reaction time was increased from 1 to 2 h. This loss was accompanied by SAR increase from 4 to 12.

### 3.6 Mineralogy of Pre‐Treated Coal Fly Ash

**FIGURE 6:**
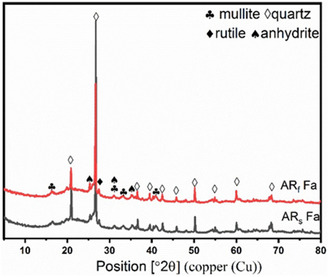
Mineralogy of pre‐treated coal fly ash.

To better understand the chemical and mineralogical transformations during acid leaching, samples treated under optimal conditions for each method were selected for analysis. For acid refluxing, the conditions were 80°C for 1.5 h with 37% HCl, while for acid roasting, samples were treated at 80°C for 1 h with the same acid concentration. The mineralogy of treated samples is shown in Figure 6, and the concentration of each mineral is presented in Table 2. As shown, the peaks for calcite (CaCO_3_) and haematite (Fe_2_O_3_) are absent in the treated samples, indicating their complete dissolution by acid, regardless of the leaching method used [12].

**TABLE 2:** Effect of acid refluxing and acid roasting treatment systems on mineral composition of coal fly ash.

Treatment	Anhydrite	Mullite	Calcite	Quartz	Rutile	Haematite
Raw FA	32.50	19.90	18.90	17.00	0.50	10.60
ARf FA	17.40	48.30	0.00	33.80	0.60	0.00
ARs FA	19.20	39.20	0.00	39.80	1.70	0.00

The disappearance of calcite (CaCO_3_) and haematite (Fe_2_O_3_) peaks in the XRD patterns of the treated samples (Table 2) indicates the complete dissolution of these specific crystalline phases by acid leaching, regardless of the method used. This is further supported by XRF analysis (Table 3), which shows a substantial reduction in the overall concentrations of CaO and Fe_2_O_3_ in the treated CFA compared to the raw material. Under optimum conditions, acid refluxing reduced Fe_2_O_3_ and CaO to 0.58% and 0.50%, respectively, while acid roasting resulted in 0.74% Fe_2_O_3_ and 1.96% CaO. These results confirm that acid refluxing achieved superior removal efficiency for both iron and calcium.

**TABLE 3:** Coal fly ash composition variation under different acid treatment systems (wt.%) [18].

Oxides, wt.%	Raw CFA (U‐CFA)	ARf CFA	Acid‐roasted CFA (ARs)
Al_2_O_3_	27.20	28.20	29.81
CaO	17.34	0.50	1.96
Fe_2_O_3_	8.08	0.58	0.74
K_2_O	0.417	0.62	0.54
Na_2_O	0.84	0.37	0.68
SiO_2_	32.86	63.20	59.97
SO_3_	8.78	0.44	0.46
TiO_2_	2.09	3.61	3.12
Total	97.60	97.51	97.28
SiO_2_/Al_2_O_3_	1.21	2.24	2.01

However, despite the near‐complete removal of calcite and haematite, the persistence of anhydrite (CaSO_4_), which decreased but remained at 17.40% and 19.20% for refluxed and roasted samples, respectively, indicates that not all calcium‐bearing phases were fully dissolved. This is consistent with previous findings that calcium may remain in more acid‐resistant forms, such as anhydrite or as part of the amorphous matrix [10, 12, 29]. Thus, while specific Fe and Ca phases were fully leached, residual amounts of these elements likely persist in more stable or less reactive forms.

The SAR also increased significantly, from 1.21 in raw CFA to 2.24 with acid refluxing and 2.01 with acid roasting, indicating improved purity and removal of impurities during the treatment process. Acid treatment of fly ash enhanced the relative concentrations of SiO_2_ and TiO_2_, as well as their associated minerals, such as quartz, mullite and rutile, as indicated in Tables 2 and 3. This apparent increase is due to the selective dissolution of other components, such as calcium and iron oxides, which leaves behind the less soluble SiO_2_ and TiO_2_ [5, 7]. The mineral composition of CFA is diverse, with iron typically present as magnetite, maghemite and/or haematite, and calcium as gypsum, anhydrite and/or calcite, all of which readily dissolve in mineral acids [10, 12, 29] Al_2_O_3_ and SiO_2_ in fly ash occur in various forms: as crystalline mullite, in an amorphous state or within an aluminosilicate glass matrix. These forms exhibit varying levels of resistance to acid leaching. The amorphous form is more susceptible to direct acid leaching, while crystalline mullite and quartz require activation for significant dissolution [5]. Mineral acids rapidly dissolve the glassy portion of fly ash, while less reactive minerals, such as mullite and quartz, remain essentially unchanged [14].

### 3.7 Characterisation of Zeolites from the Pre‐treatment of Coal Fly Ash

#### 3.7.1 Phase Composition and Chemical Composition of Synthesised Zeolites

**FIGURE 7:**
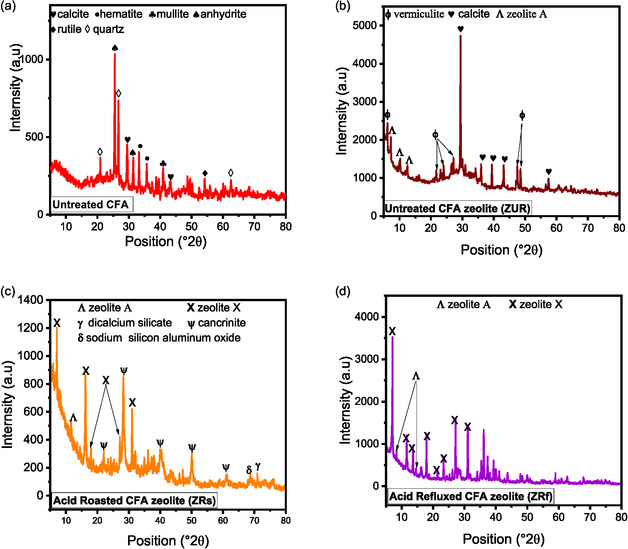
X‐ray diffraction patterns of (a) untreated CFA, (b) untreated CFA zeolite, (c) acid‐roasted CFA zeolite and (d) acid‐refluxed CFA zeolite [20].

The crystalline phases present in the synthesised zeolites were characterised by XRD (Figure 7), and their bulk chemical compositions were determined by XRF (Table 4). Zeolite synthesised from untreated CFA (ZU‐CFA) was dominated by non‐zeolitic crystalline phases, calcite (CaCO_3_) accounting for 64.4% and vermiculate with the approximate formula H_10.8_A_2.94_Ca_0.06_Fe_0.44_Mg_5.68_Na_0.04_O_27.4_Si_5.66_Ti_0.042_. Only small amounts of zeolitic phases were observed, indicating poor crystallisation. This is consistent with previous findings that high‐Ca fly ash (>5% Ca) leads to the formation of low‐value or non‐zeolitic phases such as cancrinite and hydroxysodalite [8, 9]. In contrast, zeolites synthesised from acid‐treated fly ash (Figure 7c,d) showed markedly improved crystallinity and phase purity. XRD analysis of zeolite from acid‐refluxed fly ash (ZRf) revealed a product dominated by low silica zeolite X (LSX) (84%), with minor zeolite A and no detectable calcite or vermiculite, suggesting highly effective removal of Ca and Fe. Zeolite from acid‐roasted fly ash (ZRs) showed a more complex phase composition, including cancrinite, dicalcium silicate, sodium aluminium silicate, zeolite LSX and zeolite A, indicative of incomplete impurity removal.

**TABLE 4:** The chemical composition of zeolites synthesised from Morupule coal fly ash.

OXIDE	AMOUNT, wt.%			
	U‐CFA	ZU‐CFA	ZRf	ZRs
Al_2_O_3_	27.20	21.80	27.52	28.04.
CaO	17.34	17.27	0.67	1.81
Fe_2_O_3_	8.08	7.98	0.48	0.63
K_2_O	0.42	0.15	0.43	0.25
Na_2_O	0.84	13.60	10.90	11.00
SiO_2_	32.86	35.32	55.12	51.33
SO_3_	8.78	0.12	0.02	1.78
TiO_2_	2.09	1.59	4.28	3.47
Total	97.60	97.83	99.42	98.31
SiO_2_/Al_2_O_3_	1.21	1.62	2.00	1.83

*Note:* The amount of oxide is presented in weight percentages (wt.%) as measured by XRF. U‐CFA‐ as received Morupule coal fly ash (Raw CFA), ZU‐CFA ‐zeolite from untreated coal fly ash, ZRf‐zeolite from acid‐refluxed pre‐treated coal fly ash and ZRs‐zeolite from acid‐roasted pre‐treated coal fly ash.

XRF analysis (Tables 3 and 4) supported these findings, showing that untreated CFA contained high Ca (17%) and Fe (8%), which were inherited by the synthesised ZU‐CFA product. These high impurity levels are linked to the dominant non‐zeolitic phases observed. In contrast, both acid‐treated samples (ZRs and ZRf) showed significantly reduced Ca and Fe levels (<2%), with ZRf displaying the lowest residual concentrations, correlating with its purer zeolitic composition. Elevated Na_2_O content in the synthesised zeolites was attributed to NaOH introduced during the alkaline roasting synthesis step. Sodium ions serve as structure‐directing agents, charge‐balancing agents and stabilisers within the zeolite framework [4]. The combined XRD and XRF results underscore the critical role of pre‐treatment in transforming high‐calcium CFA into suitable precursors for zeolite synthesis. High Ca and Fe contents in untreated CFA not only consume reactive silica and alumina – reducing availability for zeolite crystallisation – but also promote competing crystalline phases that physically hinder nucleation and growth [8].

Among the two methods, acid refluxing emerged as the superior pre‐treatment strategy, achieving near‐complete removal of Fe and Ca and enabling the formation of high‐purity zeolites LSX and A. These phases are desirable for ion‐exchange and water treatment applications due to their high CECs. Conversely, the presence of residual calcic phases in ZRs suggests limitations of acid roasting, particularly when dealing with high‐Ca fly ashes [8, 9, 18]. The results clearly demonstrate that the phase composition of synthesised zeolites is directly governed by the bulk chemical composition of the precursor material [4]. High Ca and Fe concentrations correlate with poor zeolite crystallisation, while pre‐treatment—particularly acid refluxing—effectively lowers these elements and promotes the formation of high‐purity zeolite X and A phases [8].

#### 3.7.2 Textural, Porosity and Structural Properties

Nitrogen adsorption–desorption analysis was conducted to evaluate the textural properties of the sample. Before measurements, the sample was degassed under vacuum at 300°C for 8 h to remove physisorbed moisture and gases. Isotherms were collected at 77 K using nitrogen as the adsorbate over a relative pressure range of *P*/*P*_0_ = 0.001–1.0. The Brunauer–Emmett–Teller (BET) surface area was calculated within the linear region (*P*/*P*_0_ = 0.006–0.03) and (*P*/*P*_0_ = 0.05–0.3), selected based on the Rouquerol criteria to ensure accuracy; the *C* constant, monolayer capacity and correlation coefficient were recorded. Micropore and external surface areas were determined using the t‐plot method. The pore‐size distribution was estimated using the Barrett–Joyner–Halenda (BJH) and Horvath–Kawazoe (HW) methods.

**FIGURE 8:**
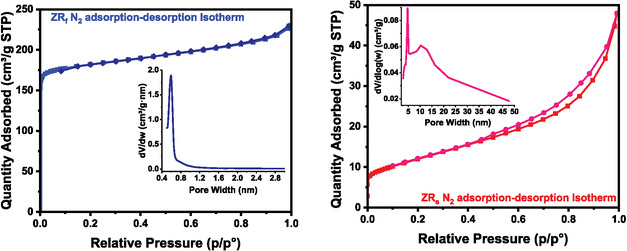
N_2_ adsorption–desorption isotherms and pore‐size distribution (sight) of zeolite synthesised from different acid pre‐treated coal fly ash protocols. Acid refluxing (left) and acid roasting (right).

Nitrogen adsorption–desorption isotherms (Figure 8 and Table 5) reveal pronounced differences in the textural characteristics of the ZRf and ZRs samples, reflecting the distinct acid treatment approaches’ impact on pore structure and zeolite crystallinity. The isotherm for ZRf shows a type I profile, which, according to IUPAC classification, is a characteristic of materials with predominant microporosity. The isotherm of ZRf shows a step uptake at low relative pressure (*P*/*P*_0_ < 0.05), followed by a well‐defined plateau with minimal further uptake [4, 30]. This indicates that the material has reached saturation (monolayer formation) at a low relative pressure; hence, the pores followed the Langmuir adsorption model for micropores. The desorption process shows a small hysteresis, which may arise from the amorphous phase forming macropores at the external surface or from interparticle void filling [23]. BET and t‐plot analyses revealed that the material possesses a high surface area of 728.8 m^2^/g (which is highly made up of micropore surface area of 632.6 m^2^/g) and a micropore volume of 0.238 cm^3^/g. This indicates that over 85% of the total surface area is attributed to micropores. This was confirmed by *H*–*K*, which showed a 0.6 nm pore‐size distribution (Figure 8 insight), consistent with the well‐known faujasite framework structure of zeolite X [30, 31]. The nearly closed hysteresis loop indicates the presence of uniform, stable micropores, often associated with well‐crystallised zeolitic frameworks. This observation aligns with the XRD results, which identify zeolite LSX as the dominant phase, along with a minor presence of zeolite A, both known for their regular pore structures and high crystallinity. The uniform microporosity arises from the ordered channels and cages inherent to these zeolites [31].

**TABLE 5:** Textural properties of synthesised zeolite from coal fly ash.

Material	Source	Method of synthesis	*S*_BET_, m^2^/g	*S*_micro_, m^2^/g	Diameter, nm	*V*_micro_, cm^3^/g	Ref.
Can/DCS/A/X	HCFA	ARfAFAH	43.03	2.52	3–20	0.06	This work
NaX	HCFA	ARsAFAH	728.81	632.61	0.60	0.24 (0.11)^b^	This work
NaX	CFA	ATAFAH	648.42	578.64	0.90	0.22	[4]
NaX	CFA	AFAH	540.09	N/A	N/A	0.19	[22]
							
NaX	CFA	UAFAH	515.52	N/A	N/A	0.18	[22]
NaX	CFA	UAFAH	486	334	1.39 (4.18)^a^	0.13 (0.17)^b^	[23]
NaX	BT	SAEH	571	449.00	0.5–0.65	0.19	[30]
NaX	Pure chemicals	H	931.00	N/A	0.61	0.40	[31]

Abbreviations: AFAH, Alkaline fusion assisted hydrothermal; ARfAFAH, acid refluxing and alkaline fusion assisted hydrothermal process; ARsAFAH, acid roasting and alkaline fusion assisted hydrothermal process; ATAFAH, acid‐treated Alkaline fusion assisted hydrothermal; BT, Bauxite tailings; Can, Cancrinite; CFA, coal fly ash; DCS, dicalcium silicate; H, hydrothermal synthesis from CFA derived Silica; HCFA, high calcium coal fly ash; *S*_BET_, Specific surface area measured by BET method; *S*_micro_, surface area contributed by micropore; UAFAH, Ultrasound‐alkaline assisted hydrothermal; *V*_micro_, micropore volume.

a mesopore.

b mesopore volume.

In contrast, the ZRs sample displays a Type IV isotherm, typically associated with mesoporous materials. It shows gradual nitrogen uptake over a broader pressure range, along with an H3‐type hysteresis loop, commonly linked to slit‐shaped pores formed between plate‐like or aggregated particle systems, which are filled via capillary condensation [31, 32, 33]. The BET surface area is considerably lower at 43.03 m^2^/g, with a microporous contribution of only 2.52 m^2^/g. The majority of the surface area (40.51 m^2^/g) is attributed to the external surface and mesoporosity, indicating a lack of significant microporosity. This is confirmed by t‐plot and BJH, which showed a negligible microporous volume (0.0008 cm^3^/g) and a broad mesoporous distribution of 3 to over 20 nm, with the main pore width approximately 3.8–4.2 nm. These findings suggest the presence of disordered or poorly crystallised material, as supported by the XRD patterns, which show a mixture of cancrinite, dicalcium silicate, zeolite LSX and a sodium aluminium silicate phase [31]. Such mixed and non‐zeolitic phases are often indicative of incomplete or disrupted zeolite synthesis. Further insight comes from the XRF data, which reveal elevated levels of residual Ca and Fe in ZRs when compared to ZRf (Table 4). These elements are known to favour the formation of non‐zeolitic calcium silicates, such as dicalcium silicate and cancrinite. Their presence likely interferes with zeolite nucleation and pore development, leading to the formation of dense or layered structures that lack microporosity and suppress crystallinity [8].

The contrasting isotherm behaviours and textural parameters underscore the critical role of the acid treatment method. Acid refluxing (ZRf) effectively removes interfering cations, such as Ca^2+^ and Fe^3+^, fostering an environment conducive to microporous zeolite crystallisation with high phase purity. In contrast, acid roasting (ZRs), though partially beneficial, fails to eliminate calcium to the same extent. The residual calcium promotes the growth of mesostructured or non‐zeolitic phases, ultimately resulting in a mesoporous‐dominated texture with low surface area and reduced crystallinity. This is in agreement with the works of Boycheva et al. [23], who reported lower zeolite X surface area with significantly large mesopore formation from CFA with higher Ca content compared to those with lower Ca. Yu et al. [31] reported a decrease in surface area when the Na^+^ ions in zeolite X were exchanged with Ca^2+^ and K^+^ ions.

It is also important to highlight that the zeolite synthesised using the acid refluxing method exhibits significantly superior textural and porosity characteristics compared to other zeolites synthesised directly from CFA reported in the literature, ranking just below the zeolite synthesised from pure chemical precursors (Table 5). This underscores the effectiveness of acid refluxing in removing calcium and iron impurities. The successful elimination of these impurities is critical, as they are known to interfere with zeolite crystallisation and framework development. These findings have far‐reaching implications. They demonstrate that with appropriate pre‐treatment, specifically acid refluxing, CFA, even of suboptimal quality, can be transformed into high‐performance zeolites suitable for advanced applications. This not only improves the economic viability of CFA valorisation but also contributes to sustainable waste management by converting an abundant industrial by‐product into value‐added materials.

**FIGURE 9:**
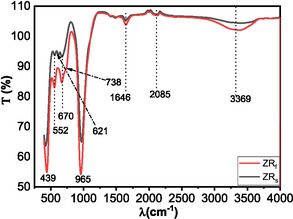
IR of zeolites synthesised from pre‐treated coal fly ashes, Zeolite ZRf (top) and Zeolite ZRs (bottom).

The zeolites synthesised from acid‐pre‐treated CFAs were also characterised using AT‐FTIR to show the functional different groups of the zeolites. The ATR‐FTIR spectra of zeolite materials synthesised from acid‐roasted and acid‐refluxed CFA show characteristic framework vibrations associated with aluminosilicate structures (Figure 9). Both samples display prominent bands around 439–470 cm^−1^ (TO bending vibrations of Si—O—Al and Si—O—Si), 552–570 cm^−1^ (double ring vibrations), and a strong asymmetric stretching band between 965–1050 cm^−1^ (Si—O—T) [4]. The broad bands 2085, 3369 and 1646 cm^−1^ are due to OH‐stretching and water molecules present in the zeolite channels. The refluxed sample shows notably sharper, more intense peaks, especially in the Si—O—T and O—H stretching regions. In contrast, the acid‐roasted sample exhibits broader, less defined peaks. A distinct band at 621 cm^−1^, linked to cancrinite, appears only in the acid‐roasted sample, indicating the presence of secondary crystalline phases, as confirmed by XRD [34, 35].

Although the refluxed sample contains a higher proportion of zeolite X, its absorption bands—particularly the Si—O—T asymmetric stretching and T—O bending modes—appear at slightly lower wavenumbers than those of the acid‐roasted sample. This shift is attributed to the higher crystallinity, uniform Si/Al distribution and purer phase composition of the refluxed fly ash zeolite, where Na^+^ is the dominant extra‐framework cation. In contrast, the acid‐roasted sample shows broader bands due to structural disorder arising from the presence of multiple crystalline phases, such as cancrinite, dicalcium silicate and sodium aluminium silicate [34, 35]. The mixed‐phase composition introduces local distortions and variable cation occupancy (Ca^2+^, Fe^3+^, Na^+^), creating non‐uniform vibrational environments that widen the absorption bands [36, 37]. These findings correlate with BET analysis, in which the refluxed sample exhibits dominant microporosity and a higher surface area, while the roasted sample shows mesoporosity and a lower microporous contribution [23].

## 4 Conclusions

This study demonstrated the effectiveness of acid refluxing and acid roasting methods for removing Fe and Ca impurities from CFA, thereby improving its suitability for zeolite synthesis. Both methods significantly reduced impurity levels, with Fe and Ca concentrations dropping to below 2%. Acid refluxing achieved superior removal efficiencies of 99% for Ca and 97% for Fe under optimised conditions (85°C, 1.5 h, 37% HCl). Acid roasting, while effective, was slightly less efficient, achieving 95% removal of both impurities under its optimal conditions (80°C, 1 h, 37% HCl). Additionally, acid refluxing enhanced the SAR more effectively, indicating better impurity removal and material purity. This is further confirmed by the production of a purer zeolite X from acid‐refluxed fly ash as compared to acid‐roasted and untreated CFA, which had low crystalline products with Ca mineral impurities. BET analysis further revealed that zeolite synthesised from refluxed CFA possessed a high surface area (728.8 m^2^/g) and uniform microporosity (∼0.6 nm). In contrast, acid roasting‐derived zeolite was mesoporous (3–20 nm) with a lower surface area (48 m^2^/g). These structural distinctions underline the impact of the pre‐treatment method on zeolite quality. Based on these findings, acid refluxing is recommended as the preferred pre‐treatment method for preparing CFA for high‐quality zeolite synthesis. This approach not only enhances the reactivity of CFA but also supports sustainable waste management and the production of valuable materials.

## Author Contributions

**Emmanuel Monkgomotsi Victor Gaolefufa:** conceptualisation and methodology, formal analysis and investigation, writing ‐ original draft preparation. **Thapelo Manyapedza:** formal analysis and investigation. **Bakang F. Modukanele:** formal analysis and investigation. **Moses T. Kabomo:** writing ‐ review and editing, supervision. **Isaac N. Beas:** Writing ‐ review and editing, supervision. All authors read and approved the final manuscript.

## Funding

The authors have nothing to report.

## Conflicts of Interest

The authors declare no conflicts of interest.

## Data Availability

Data available upon reasonable request.
